# Alcohol Policy in Adolescence and Subsequent Alcohol-attributable Hospitalizations and Mortality at Ages 21−54 Years: A Register-based Cohort Study

**DOI:** 10.1097/EDE.0000000000001857

**Published:** 2025-04-04

**Authors:** Juha Luukkonen, Elina Einiö, Lasse Tarkiainen, Pekka Martikainen, Hanna Remes

**Affiliations:** From the a Helsinki Institute for Demography and Population Health, University of Helsinki, Helsinki, Finland; b Max Planck–University of Helsinki Center for Social Inequalities in Population Health, Helsinki, Finland.

**Keywords:** Adolescent drinking, Alcohol policy, Alcohol-attributable hospitalizations, Alcohol-attributable mortality, Chronic alcohol-attributable diseases, External-cause mortality, Long-term health

## Abstract

**Background:**

Little is known about how alcohol policies experienced in adolescence are associated with later health. We assess whether the age of exposure to stricter alcohol policies is associated with later alcohol-attributable hospitalizations and mortality. We take advantage of an alcohol advertising ban and alcohol tax increases introduced in 1975–1977 with relatively stable alcohol policies before and after.

**Methods:**

We used Finnish register data on birth cohorts 1950–1964 (1,175,878 individuals) to assess cohort-wise hazard ratios for the first incidence of alcohol-attributable hospitalization and mortality, and mortality due to external and other causes at ages 21–54 years.

**Results:**

Men who were aged 19–25 at the time of the restrictive reform had similar risks for alcohol-attributable hospitalization and mortality to the reference group of those aged 18—legal drinking age—at the time of reform. For those underage at the time, hospitalization and mortality rates were incrementally smaller cohort by cohort. For example, men who were 17 at the time of the reform had lower hazard ratios of alcohol-attributable hospitalization: 0.91 (95% confidence interval: 0.87, 0.95) as did those who were 13 (0.85; 95% confidence interval: 0.81, 0.89). The findings were similar for external-cause mortality, and similar yet more uncertain for women. In contrast, mortality from other causes declined continuously from cohort to cohort.

**Conclusions:**

Our findings are consistent with the hypothesis that stricter alcohol policies in adolescence reduce harmful alcohol consumption patterns extending into adulthood and manifesting as lower alcohol-related harm to health.

Early initiation of alcohol use is a powerful predictor of later use in terms of quantity consumed and adverse drinking habits adopted.^1,2^ In particular, heavy alcohol use in adolescence may disrupt brain development and create fertile ground for substance use disorders.^3,4^ Thus, one could consider alcohol policies that target youth consumption to be especially effective in reducing alcohol-related harm. However, the evidence of how exposure to alcohol policies in youth, particularly in adolescence when alcohol use is often initiated,^5,6^ may shape health in the long term is scarce.

Stringent alcohol policy environments in the US, Canada, Australia, and Europe have been associated with both less drinking and less hazardous drinking patterns for youth.^7,8^ However, causal inference from cross-sectional studies assessing associations between alcohol policies and alcohol consumption in between-country and other area comparisons is difficult since both policies and consumption can reflect long-standing cultural factors and attitudes related to alcohol consumption.^9^ In this sense, studies focusing on changing policies can provide more reliable information on policy impacts. Within-country studies on changes in alcohol policies and immediate youth consumption responses provide mixed findings depending on the alcohol policy in question. For example, while increasing the legal drinking age has been shown to reduce youth alcohol consumption,^10^ the evidence on whether and how changes in alcohol prices affect youth alcohol consumption is weak. Reviews focusing on prices and taxation interventions found a lack of consistent results between alcohol prices and youth drinking,^11,12^ including four studies from Finland reporting no increased youth drinking after a significant alcohol tax cut in 2004.^11^ Furthermore, another review found that heavy episodic drinkers were generally non-responsive to increased alcohol prices, and this nonresponsiveness held for binge drinkers in all population subgroups, including the youth.^13^ Finally, a recent Swedish study found that both minimum and average alcohol prices were not associated with current drinking of 15–16-year-olds in 1989–2017.^14^

Existing evidence on alcohol advertising and youth drinking is mixed,^15^ often attributed to difficulties in measuring the exposure.^15,16^ Two systematic reviews focusing on alcohol advertising or media exposure of youth have found that being exposed to alcohol advertising and promotion is associated with an increased likelihood of adolescent drinking in terms of initiation of alcohol use, the quantity of alcohol consumed, and more hazardous drinking patterns.^17,18^ However, the included survey-based studies rely on highly heterogeneous and specific measures of advertisement and media exposures,^17,18^ which are not actual interventions in the sense that exposure to alcohol advertising could potentially be determined by confounding factors, such as familial attitudes towards alcohol use.^17^ Conversely, an econometric review found highly mixed evidence on how alcohol advertising is associated with consumption, with some newer studies supporting that exposure to alcohol advertising slightly increases youth alcohol consumption.^19^

Overall, evidence on the short-term impact of changes in alcohol prices and advertising on current drinking among youth is unclear or modest, perhaps due to the known difficulties in obtaining a reliable measurement of alcohol consumption related to reporting bias and nonresponse in surveys.^20,21^ Moreover, as alcohol-related chronic diseases take years to develop, and thus tend to occur only at later ages, a longer perspective in assessing the impact of alcohol policies experienced in youth is warranted. Currently, evidence on how alcohol policies experienced in youth may be associated with long-term health outcomes is scarce and limited to studies assessing changes in the minimum legal drinking age. These studies have provided comprehensive evidence on how being legally able to purchase alcohol at younger ages is associated with increased rates of later adulthood binge drinking,^22^ as well as increased chronic alcohol-attributable morbidity and mortality rates even decades later.^23,24^ Besides these few studies on legal drinking age, there have been no efforts to assess how exposure to different alcohol control policies in youth is associated with long-term health outcomes. We aim to fill this gap in research by means of a 3-decade follow-up among cohorts exposed to either more liberal or stricter alcohol policies in their adolescence. The Finnish alcohol policy landscape over time enables this kind of long-term scrutiny with the liberalization of alcohol policy in 1969, followed by alcohol tax increases and an advertising ban in 1975–1977, and relatively stable alcohol policies for the following two decades.

In this study, we assess whether the age of exposure to stricter alcohol policies is associated with later alcohol-attributable health outcomes, with the theoretical premise that earlier exposure to more expensive alcohol and less alcohol advertising reduces harmful alcohol consumption patterns and later-life alcohol-related harm. We compare the first incidence of alcohol-attributable hospitalizations and mortality of birth cohorts 1950–1964 during ages 21–54 years (in the period 1971–2018). In other words, we assess whether the long-term alcohol-attributable health outcomes among more recent cohorts who were younger when the stricter policies were implemented differ from earlier birth cohorts who were more likely to have already initiated alcohol use by the time of the reform.

We also assess cohort differences in external-cause mortality, since alcohol use is a common risk factor for accidents and injuries, and all other causes of death to compare these results to trends in overall mortality. Study context and policy environment are as follows: In 1969, the Finnish alcohol policy reform relaxed numerous previously strict alcohol policies, including a lowering of the minimum legal drinking age from 21 to 18 and abolishing restrictions concerning alcohol sales in rural regions.^24^ Consequently, per capita alcohol consumption grew rapidly in the following years.

To combat the ever-increasing alcohol consumption that followed these changes, the Finnish government introduced alcohol tax increases and a total advertising ban on all alcohol products.^25,26^ The government increased the alcohol tax by 28.5% in 1975, with a further 6.1% increase in 1976.^27^ The advertising ban of 1977 was near-total, exempting advertising in government monopoly stores, bars and restaurants, and professional publications aimed for the hospitality industry. All other forms of advertisement were forbidden, including product placements.^28^

However, because of rampant inflation in 1975 and government sluggishness to allow the Finnish alcohol monopoly to further raise their prices after the initial increase, the target price level was only reached from the start of 1976.^25^ By 1977, the per capita alcohol consumption stabilized for 10 years.^29^ Afterward, there were no major alcohol policy changes until Finland joined the European Union in 1995.

## METHODS

### Data

We included birth cohorts from 1950 to 1964 identified from the 1975 census, covering the total population. The census data—containing information on age, gender (defined by legal gender) and regions of birth and residence—was supplemented with information from the Care Register for Healthcare^30^ and the Cause-of-Death Register,^31^ maintained by the Finnish Institute of Health and Welfare and Statistics Finland, respectively. The study has been approved by Statistics Finland Board of Statistical Ethics (TK-53-1490-18) and the Social and Health Data Authority Findata (THL/2180/14.02.00/2020). Statistic Finland linked the census and registered data using the personal identification codes assigned to all permanent residents in Finland.^32^ The registers contain nuanced information on alcohol-attributable causes of death and inpatient hospital care episodes starting from 1971. The hospital records contain all inpatient care episodes in Finland. The cause of death was missing for 1.7% of the deceased, mainly among those who had died abroad.

The alcohol-attributable causes of death and hospitalizations included diseases that were directly attributable to alcohol, such as alcoholic liver cirrhosis or alcoholic pancreatic diseases; mental and behavioral conditions affected by alcohol use, such as alcoholism; and accidental alcohol poisoning. Nearly all deaths and 97.8% of the hospitalizations in our data are chronic alcohol-attributable illnesses. The alcohol-attributable deaths were identified with the underlying cause of death, and the alcohol-attributable hospitalizations were identified using both primary and secondary diagnoses according to data availability for the whole observation period. eTable 1; http://links.lww.com/EDE/C238 contains the International Classification of Diseases codes we used to identify alcohol-attributable deaths and inpatient hospital care episodes. We identified external causes of death with the underlying cause of death being a suicide, violent, or accidental cause, including cases where alcohol was a contributing factor but not the underlying cause of death (i.e., excluding accidental alcohol poisoning). To compare alcohol-attributable and external mortality to overall mortality development, we also assessed the group of all other causes of death, such as cardiovascular diseases, cancers, and respiratory illnesses. While most of the causes of death are based on clinical data, autopsies are routinely performed in less clear or sudden cases of death, including all suspected poisonings.^33^ The Finnish mortality statistics have been assessed as reliable,^34^ and due to the high medico-legal autopsy rates, the Finnish death certificates have been assessed to record alcohol-related deaths rather accurately.^35^

We chose the birth cohorts of 1950–1964 to compare cohorts who differed in their age of exposure to the stricter alcohol policy implemented in 1975–1977 and to ensure a long, symmetric follow-up period for later alcohol-attributable health outcomes at ages 21–54. Due to data availability, we start the follow-up at the age of 21 years; however, the incidence of alcohol-attributable hospitalizations and deaths is very low below that age.^36,37^ We used the 1957 cohort as the reference group since they reached the legal drinking age of 18 years around the time of reform. eTable 2; http://links.lww.com/EDE/C238 shows the study cohorts’ exposure to alcohol policies at each age.

### Statistical Analyses

We follow all the cohorts at ages 21–54 (in 1971–2018, depending on the birth cohort). First, we report crude alcohol-attributable hospitalization and mortality rates by cohort and gender, as well as age in three broad age groups (21–31, 32–42, and 43–54) to assess potential differences in the associations across the follow-up. We also estimate Kaplan–Meier survival curves for first alcohol-attributable hospitalization or death across ages 21–54 by cohort (eAppendices 1 and 2; http://links.lww.com/EDE/C238). Next, we perform Cox regressions with age as the underlying time scale to assess the association of the birth cohort and the two alcohol-attributable outcomes. We also assess cohort-wise differences in external mortality as well as mortality due to causes other than alcohol or external reasons. The analyses are performed separately for men and women and for each outcome, with the municipality of birth as a strata to address local differences in alcohol-attributable baseline hazards.^38^ Finally, we conducted two sensitivity analyses, the first one including even earlier cohorts 1944–1949 to assess longer term cohort-wise trends in alcohol-attributable outcomes (eAppendix 3; http://links.lww.com/EDE/C238) and the second one excluding individuals affected by an educational reform in 1972–1977 as extended schooling might have improved their later-life health (eAppendix 4; http://links.lww.com/EDE/C238).

**FIGURE 1.**
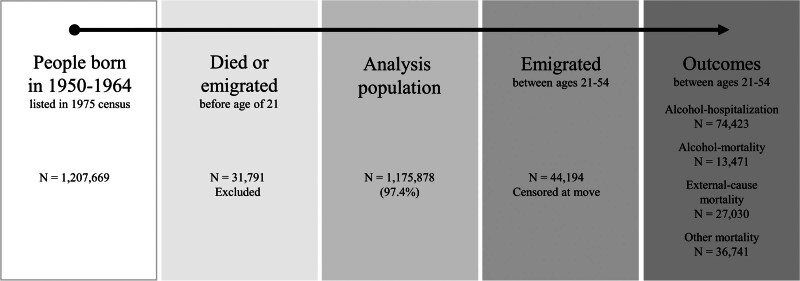
Recruitment flowchart for the study population.

We censored individuals at the age of 54 years, the time of emigration (N = 44,194), time of death (N = 77,242), or their first alcohol-attributable hospitalization in the respective analyses. Figure 1 presents the flowchart of the final study population consisting of 1,175,878 individuals.

## RESULTS

For men, alcohol-attributable hospitalization and mortality incidence rates at ages 21–54 were lower for the more recent cohorts as compared to earlier cohorts 1950–1957, who were exposed to liberal alcohol policy through their adolescence and mostly reached the legal drinking age of 18 before stricter policies took effect in 1975–1977 (Table). For men, the alcohol-attributable hospitalization rates between ages 21 and 54 were stable with around 100 hospitalizations per 1000 persons for the 1950–1957 cohorts, ranging from 102 to 95. The alcohol-attributable hospitalization rates began to gradually decline for the more recent cohorts; for example, 89 and 83 hospitalizations per 1000 persons for the 1958 and the 1962 cohort who were aged 17 and 13, respectively, at the time of reform. The mortality rates showed a similar pattern of stagnation among the earlier cohorts and a gradual decline among the more recent cohorts.

**TABLE.** Alcohol-attributable Hospitalizations and Mortality Rates Per 1000 Persons by Birth Cohort and Gender at Ages 21–31, 32–42, 43–54, and 21–54

Alcohol-attributable Hospitalizations					Men							Women		
Cohort	Age at Reform	N	Cases	21–31	32–42	43–54	21–54	Age at Reform	N	Cases	21–31	32–42	43–54	21–54
1950	25	43,783	4,435	24	47	65	99	25	40,876	1,197	6	12	20	29
1951	24	42,036	4,140	25	48	64	99	24	39,100	1,110	5	11	19	28
1952	23	43,169	4,289	24	49	64	99	23	40,311	1,195	6	12	20	30
1953	22	41,268	4,197	24	50	66	102	22	38,864	1,154	6	12	20	30
1954	21	40,997	4,085	24	48	64	100	21	39,454	1,217	6	13	21	31
1955	20	41,184	4,099	23	50	63	100	20	38,846	1,239	6	14	21	32
1956	19	41,364	3,936	22	47	62	95	19	39,055	1,226	5	14	21	31
1957	18	40,017	3,908	23	47	63	98	18	37,726	1,174	6	14	21	31
1958	17	37,831	3,383	22	43	56	89	17	35,958	1,102	6	13	20	31
1959	16	38,850	3,461	21	45	57	89	16	37,026	1,072	5	13	20	29
1960	15	38,784	3,322	21	41	54	86	15	36,891	1,114	6	13	20	30
1961	14	38,617	3,141	20	39	52	81	14	37,026	1,055	5	12	18	29
1962	13	38,769	3,200	20	38	54	83	13	36,931	986	6	12	17	27
1963	12	38,968	3,026	19	37	49	78	12	37,253	926	5	11	16	25
1964	11	38,293	2,883	19	35	47	75	11	36,631	979	6	13	16	27
Total		603,930	55,505	22	44	59	92		571,948	16,746	6	13	19	29
Alcohol-attributable Mortality					Men							Women		
Cohort	Age at Reform	N	Cases	21–31	32–42	43–54	21–54	Age at Reform	N	Cases	21–31^a^	32–42	43–54	21–54
1950	25	43,783	755	1	5	12	17	25	40 876	141	0	1	3	3
1951	24	42,036	737	1	4	13	18	24	39 100	155	0	1	3	4
1952	23	43,169	774	1	4	13	18	23	40 311	154	0	1	3	4
1953	22	41,	739	1	5	12	18	22	38 864	183	0	1	4	5
1954	21	40,997	731	1	4	13	18	21	39 454	158	0	1	3	4
1955	20	41,184	769	1	4	14	19	20	38 846	174	0	1	4	5
1956	19	41,364	744	1	4	13	18	19	39 055	196	0	1	4	5
1957	18	40,017	701	1	5	12	18	18	37 726	183	0	1	4	5
1958	17	37,831	646	1	4	12	17	17	35 958	174	0	1	4	5
1959	16	38,850	643	1	4	12	17	16	37,026	165	0	1	4	4
1960	15	38,784	645	1	4	12	17	15	36,891	160	0	1	3	4
1961	14	38,617	558	1	3	11	14	14	37,026	137	0	1	3	4
1962	13	38,769	593	1	4	11	15	13	36,931	139	0	1	3	4
1963	12	38,968	536	1	3	10	14	12	37,253	129	0	1	2	4
1964	11	38,293	545	1	4	10	14	11	36,631	148	0	1	3	4
Total		603,930	10,116	1	4	12	17		571,948	2,396	0	1	3	4

Full population, Finnish birth cohorts 1950–1964.

a For women, the alcohol-attributable deaths between ages 21 and 31 ranged from 1 to 10 cases by cohort.

The alcohol-attributable outcomes of women were a fraction of that of men’s. The hospitalization rates ranged from 28 to 32 cases per 1000 persons for the earlier cohorts 1950–1957, with a slight decline for the more recent cohorts (around 29–25 hospitalizations per 1000 persons). In general, the alcohol-attributable hospitalization and mortality rates increased by age, but the hospitalization rates exhibited similar cohort differences across the follow-up (see eAppendices 1 and 2; http://links.lww.com/EDE/C238 for the respective Kaplan–Meier survival curves), while the differences in mortality rates between cohorts emerged at later ages.

**FIGURE 2.**
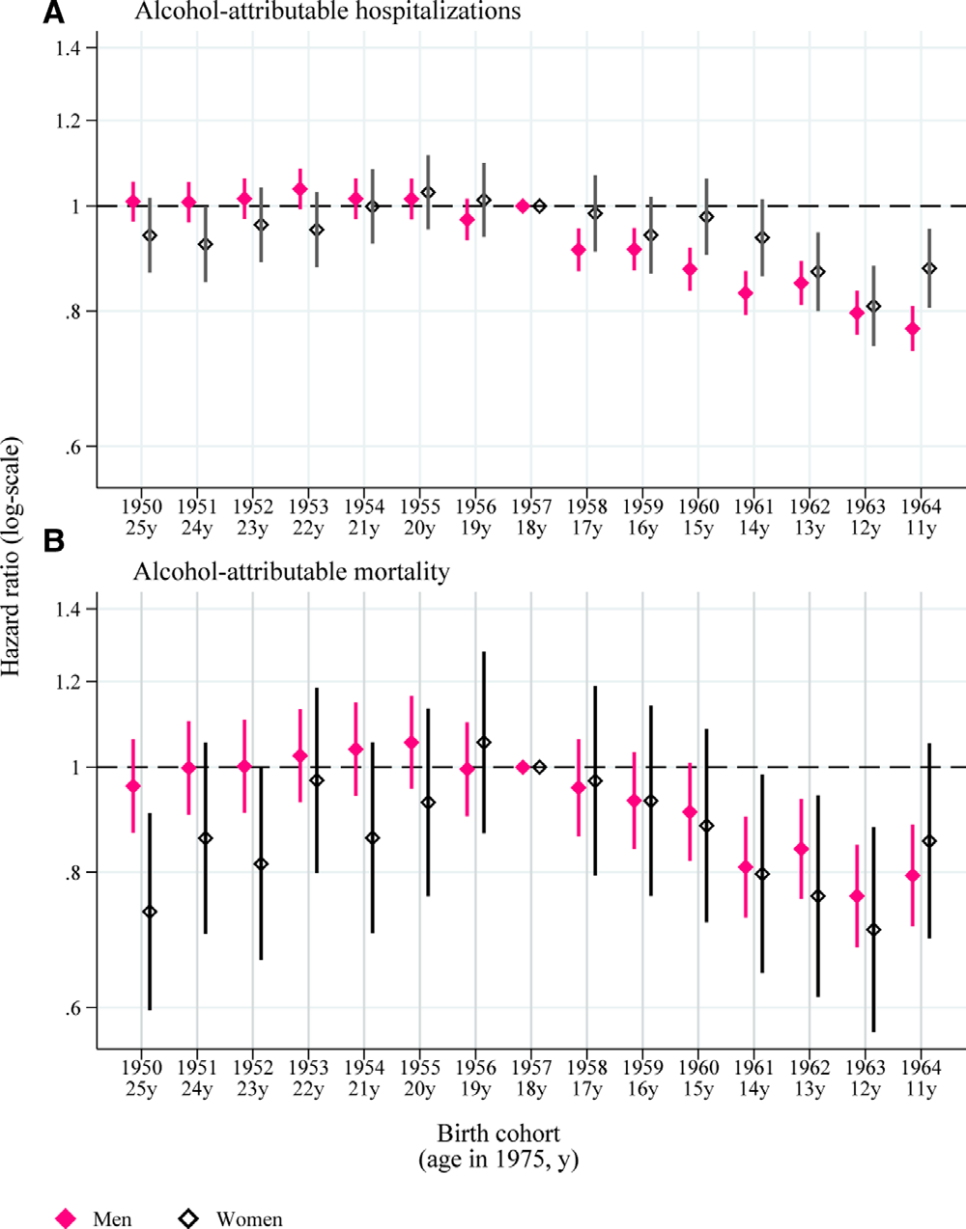
Hazard ratios with 95% confidence intervals for the first incidence of alcohol-attributable (A) hospitalization and (B) mortality between ages 21 and 54 years by birth cohort (and age in 1975 when the stricter alcohol policies were implemented). Reference category 1957 cohort (18 years).

For men, the earlier-born cohorts had a similar risk of hospitalization or death due to alcohol-attributable causes as the reference cohort of 1957 (Figure 2) in survival models adjusted for age and area. In contrast, the hospitalization hazard ratios (HR) of the more recent cohorts get incrementally smaller, starting with 0.91 (95% confidence interval [CI]: 0.87, 0.95) of the 1958 cohort to 0.77 (95% CI: 0.73, 0.81) of the 1962 cohort, and the respective mortality HRs are 0.96 (95% CI: 0.86, 1.06) and 0.85 (95% CI: 0.81, 0.89). For women, the earlier cohorts exhibit a slightly increasing trend in alcohol-attributable harm that stagnated around the 1957 cohort and started to decline with the more recent cohorts, but the CIs are wider than with men.

**FIGURE 3.**
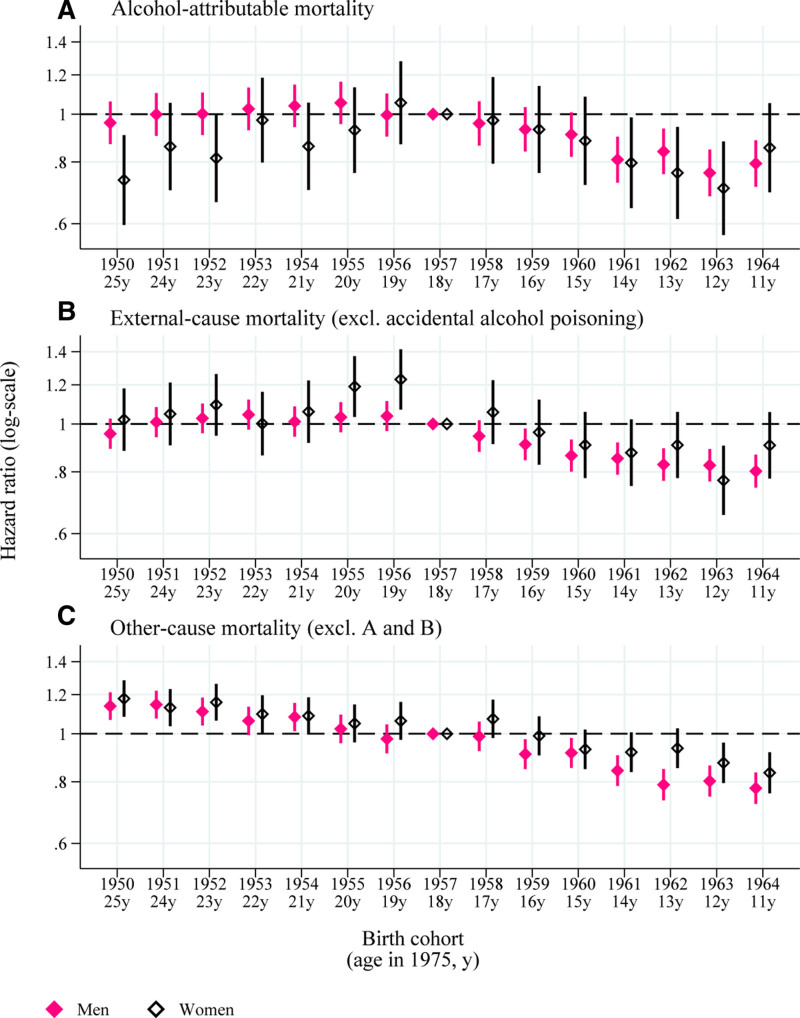
Hazard ratios with 95% confidence intervals for (A) alcohol-attributable, (B) external-cause, and (C) other-cause mortality for men and women between ages 21 and 54 years by birth cohort (and age in 1975 when the stricter alcohol policies were implemented). Reference category 1957 cohort (18 years). Accidental alcohol poisonings have been excluded from external-cause mortality.

The external-cause mortality, excluding accidental alcohol poisonings, follows alcohol-attributable mortality closely for both men and women, and similar cohort trends for long-term external-cause mortality can be observed as with alcohol-attributable mortality (Figure 3). Especially with men, the external-cause mortality development resembles the alcohol-attributable mortality, with the HRs of earlier-born cohorts not differing from the reference cohort of 1957. For example, the HR for 1950 was 0.96 (95% CI: 0.89, 1.03) and for 1955 was 1.03 (95% CI: 0.97, 1.06). As with alcohol-attributable mortality, the external cause of death HRs of the more recent cohorts became incrementally smaller, starting with 0.95 (95% CI: 0.88, 1.02) of the 1958 cohort to 0.83 (95% CI: 0.77, 0.89) of the 1962 cohort. For women, the CIs for external-cause mortality mostly overlapped with the reference cohort but were generally similar in shape as with alcohol-attributable hospitalizations and mortality: first rather stable for earlier cohorts and incrementally decreasing for the more recent cohorts.

Figure 3 also shows the differences in mortality due to causes other than alcohol-attributable and external causes. The mortality due to other causes between ages 21 and 54 gradually decreased cohort by cohort for both men and women.

## DISCUSSION

Using Finnish population register data, we assessed how the age of exposure to stricter alcohol policies was associated with later-life alcohol-attributable hospitalizations and mortality and external-cause mortality up to the age of 54 years. The premise was that being exposed to more expensive alcohol and less alcohol advertising at an earlier age would be beneficial for later health outcomes. For men below the legal drinking age when the stricter policies were implemented, all of the aforementioned outcomes decreased cohort by cohort. Conversely, alcohol-attributable harms were on a stably higher level for the earlier cohorts, who were aged 19–25 years at the time of the reform and more likely to have already initiated alcohol use.

For women, the results were less conclusive due to the outcomes being rare at ages 21–54, albeit a similar decline in the hazards of alcohol-attributable harm and external-cause mortality could be observed as with men. As alcohol-related health harms further increase by age,^37^ a prolonged follow-up period could yield more robust associations, as shown by a study assessing an earlier change in the Finnish minimum legal drinking age.^24^

The observed simultaneous changes in alcohol-attributable and external-cause mortality trends appear to be independent from the general trend of linearly declining other-cause mortality. This makes the stringent policy changes a plausible driver of this development. The external-cause mortality development also supports the policy inference since alcohol has been strongly associated with accidents, violent victimization, and self-harm in Finland.^39–41^

For men, there were no notable differences between the cohorts who were over 18 years old when the stricter policies took place, which could suggest that, despite their still young age, the restrictive policies had no impact on those who had reached the legal drinking age before the reform. The incremental decline in the cohort-wise alcohol-attributable harm after the policy change suggests that exposure to stricter policies already matters for persons who are below the drinking age. In fact, we estimated the policies to be more effective the younger the persons were at the time of reform, consistent with the hypothesis that alcohol exposure at younger ages could be more detrimental. However, the cohort comparison cannot distinguish between the potential roles between the age of exposure or cumulative amount of exposure to stricter policies, but the age of exposure does seem to play a role, as we observed stable risks for alcohol-attributable harm for the cohorts who were legal adults by the time of the reform.

Survey-based drinking habit studies corroborate our findings in showing changing drinking habits among young people in the 1970s and early 1980s. The proportion of abstainers among people aged 15–29 years old notably increased from 1976 to 1984,^42^ and the share of minors drinking at least once per month was notably less frequent in 1979 than 1973.^38^

Our results support the hypothesis that alcohol policies experienced in adolescence are potentially crucial in determining later-life health, a finding in line with prior evidence of adolescence being an especially vulnerable period for initiating addictive behaviors.^3,4^ As compared to other studies assessing associations with minimum legal drinking age and long-term alcohol-related health outcomes,^23,24^ the results are similar in that stricter policies around the time of reaching the legal drinking age were protective from later-life alcohol-attributable harm. To further show how the earlier liberalization of Finnish alcohol policies was associated with the later health of adolescents exposed to them, we expanded the inspection to birth cohorts 1944–1964, whom we could follow between ages 27 and 54. eAppendix 3; http://links.lww.com/EDE/C238 shows survival analysis results on later alcohol-attributable harm for cohorts exposed to the 1969 liberalization of alcohol policies and for cohorts exposed to stricter policies from 1975 onwards. The figure further demonstrates that more restrictive alcohol policies at the time of adolescence were associated with less alcohol-attributable harm, which is observable for both the earliest cohorts who were already adults when the liberal reform of 1969 was implemented and for the most recent cohorts who were exposed to the later stricter policies.

### Increased Alcohol Taxation Versus the Advertising Ban

Alcohol policies are often implemented in clusters, and the simultaneity of different types of policy changes makes it hard to decompose drivers behind any consequent health development.^43^ Unfortunately, this is also the case with our study since the tax increases and advertising ban were implemented in close proximity to one another.

However, based on the results of prior studies and alcohol price development in the 1970s, we believe the advertising ban to be a more likely driver of these results, although it must be noted that alcohol advertising eventually took different, more subtle forms.^26^ In prior studies, advertising exposure has been hard to piece together in terms of its omnipresent nature, and might be the reason behind the modest results regarding many specific advertising exposure metrics.^15^ Cumulative advertising exposure is also difficult to assess by age, as being exposed to advertising in earlier or at different ages might have different implications regarding the individual,^44^ and for example, once positive expectations of alcohol have been formed at an early age, later restrictions to alcohol advertising might be less impactful.^16^ This might well explain why advertisement bans have not been associated with population-level total alcohol consumption,^45,46^ with the exception of Norway, in which after an advertising ban was implemented in 1975, the increasing trend of alcohol sales ceased.^47^

Essentially our results are in line with the views that advertising restrictions slow down the recruitment of new drinkers^48^ and that especially early exposure to advertising is more impactful.^16^ While tax increases might have had some impact on the findings, in practice inflation eroded these increases in a fast pace, and the prices of beer and liquor were relatively low and stable in real terms for most of the 1970s and 1980s.^49^ Furthermore, according to prior international evidence, youth alcohol consumption does not appear to be sensitive to changes in alcohol prices,^14,50^ and the youth have been observed to have difficulties in perceiving changes in alcohol prices if they are not substantial.^51^

### Strengths and Limitations

Prior evidence suggests that the short-term impact of changes in alcohol prices and advertising on current adolescent drinking is unclear or modest. Given the difficulties in measuring alcohol consumption in adolescence and that alcohol-related harm to health tends to occur at later ages, a longer perspective in assessing their impact is warranted. With our total population data covering several decades of reliable outcome data, we have estimated such long-term associations, but there are some methodologic considerations that must be kept in mind when interpreting our findings.

The incremental cohort-wise decline in alcohol-attributable harm for the cohorts exposed to the more stringent policy environment in adolescence is solid descriptive evidence. However, the association would be more convincingly established with an actual counterfactual control group that would not experience the policy changes at all. For example, regional differences in policy implementation could provide the kind of treatment variation required for a difference-in-differences analysis. In Finland, changes in alcohol policies were not regionally phased in, thus, evidence from other policy settings are called for. After the stringent policy measures of 1975–1977, there were no major alcohol political reforms for a long period, making the analyses of these cohorts independent of other potentially confounding alcohol policy changes. However, there were also other, partly concurrent policy changes happening in the 1970s that could have affected our results. From 1972 to 1977, an educational reform was implemented stepwise in different regions of Finland.^52^ The reform could potentially have benefited the more recent cohorts also exposed to the stricter alcohol policies, so we performed a sensitivity analysis in which we identified and excluded the individuals affected by this educational reform in their municipality of residence by 1975 (N = 106,713; 9.1% of the study population) (eAppendix 4; http://links.lww.com/EDE/C238). Our results concerning the associations of the alcohol policy reform were not affected by this exclusion.

Additionally, in 1977, the implementation of the Tobacco Control Act, which introduced an age limit of 16 years for purchasing tobacco products,^53^ could also have positively affected the health of the more recent cohorts. Survey-based cohort trends have shown that a decreasing overall smoking trend was reinforced at the time the age limit was implemented.^54^ However, smoking-related morbidity usually manifests at older ages than alcohol-related mortality in the forms of cancers as well as chronic respiratory and cardiovascular diseases.^55^

Finally, other factors in which the cohorts differed by age of exposure, such as economic fluctuations or later alcohol policy changes, could have contributed to these findings. However, in the Kaplan–Meier curves (eAppendices 1 and 2; http://links.lww.com/EDE/C238), there were no major shifts in the estimated outcome incidence in later years, suggesting that later-life conditions did not explain these cohort differences.

## CONCLUSIONS

In general, studies about policy changes can provide the most valuable evidence of how the policies work in practice.^56^ We assessed how the age of exposure to stricter alcohol policies in the form of tax increases and advertising ban was associated with later-life alcohol-attributable health outcomes up to the age of 54 years. Our findings show that more strict alcohol policies at vulnerable ages were associated with decreased long-term alcohol-attributable hospitalizations and mortality, as well as with mortality due to external causes. While the results were more robust among men, similar estimates of decreases in alcohol-attributable harm and external-cause mortality were also observed among women. Our results are consistent with the hypothesis that alcohol control policies experienced in adolescence may leave a permanent imprint on alcohol consumption patterns and have substantial health implications in adulthood.

## Supplementary Material
