# Supplementary material for: Alcohol Policy in Adolescence and Subsequent Alcohol-attributable Hospitalizations and Mortality at Ages 21−54 Years: A Register-based Cohort Study
Source: Epidemiology. 2025 Apr 4;36(4):580–9. doi: 10.1097/EDE.0000000000001857 (PMC12118618; doi:10.1097/EDE.0000000000001857)
Supplement: Supplementary file 1 [file ede-36-580-s001.pdf]

eTable 1 *ICD-codes used to identify alcohol-attributable mortality and hospitalizations during 1971-2018.*

1971-1986 (ICD-8)	
291	Alcoholic psychosis
303	Alcoholism
571	Cirrhosis of the liver
980	Toxic effects of alcohol
577	Diseases of the pancreas
E860	Accidental alcohol poisoning

1987-1995 (ICD-9)	
291	Alcohol-induced mental disorders
303	Alcohol dependence syndrome
3050	Alcohol abuse
3575	Alcoholic polyneuropathy
4255	Alcoholic cardiomyopathy
5353	Alcoholic gastritis
5710	Alcoholic fatty liver
5711	Alcoholic hepatitis
5712	Alcoholic cirrhosis of liver
5713	Alcoholic liver damage, unspecified
5770D-F; 5771C-D	Alcoholic diseases of pancreas
E860	Accidental alcohol poisoning

1996-2018 (ICD-10)	
E244	Alcohol-induced pseudo-Cushing's syndrome
F10	Mental and behavioural disorders due to use of alcohol
G312	Degeneration of nervous system due to alcohol
G4051	Alcoholic epilepsy
G621	Alcoholic polyneuropathy
G721	Alcoholic myopathy
I426	Alcoholic cardiomyopathy
K292	Alcoholic gastritis
K70	Alcoholic liver disease
K852	Alcohol induced acute pancreatitis
K860	Alcohol-induced chronic pancreatitis
T51	Toxic effect of alcohol
X45	Accidental poisoning by and exposure to alcohol
Y90-91	Evidence of alcohol involvement

eTable 2 *Diagram of study birth cohorts' exposure to alcohol policies at different ages. Minimum legal drinking age 18 years.*

		Liberal alcohol policy 1969-1975							Stricter alcohol policy 1976-						
Birth cohort	1968	1969	1970	1971	1972	1973	1974	1975	1976	1977	1978	1979	1980	1981	1982
1950	18	19	20	21	22	23	24	25	26	27	28	29	30	31	32
1951	17	18	19	20	21	22	23	24	25	26	27	28	29	30	31
1952	16	17	18	19	20	21	22	23	24	25	26	27	28	29	30
1953	15	16	17	18	19	20	21	22	23	24	25	26	27	28	29
1954	14	15	16	17	18	19	20	21	22	23	24	25	26	27	28
1955	13	14	15	16	17	18	19	20	21	22	23	24	25	26	27
1956	12	13	14	15	16	17	18	19	20	21	22	23	24	25	26
1957	11	12	13	14	15	16	17	18	19	20	21	22	23	24	25
1958	10	11	12	13	14	15	16	17	18	19	20	21	22	23	24
1959	9	10	11	12	13	14	15	16	17	18	19	20	21	22	23
1960	8	9	10	11	12	13	14	15	16	17	18	19	20	21	22
1961	7	8	9	10	11	12	13	14	15	16	17	18	19	20	21
1962	6	7	8	9	10	11	12	13	14	15	16	17	18	19	20
1963	5	6	7	8	9	10	11	12	13	14	15	16	17	18	19
1964	4	5	6	7	8	9	10	11	12	13	14	15	16	17	18

Year

eAppendix 1 *Kaplan-Meier survival curves for first alcohol-attributable hospitalization by gender and birth cohort (1950-1964) during ages 21 to 54 years. Different y-axis scales for men and women.*

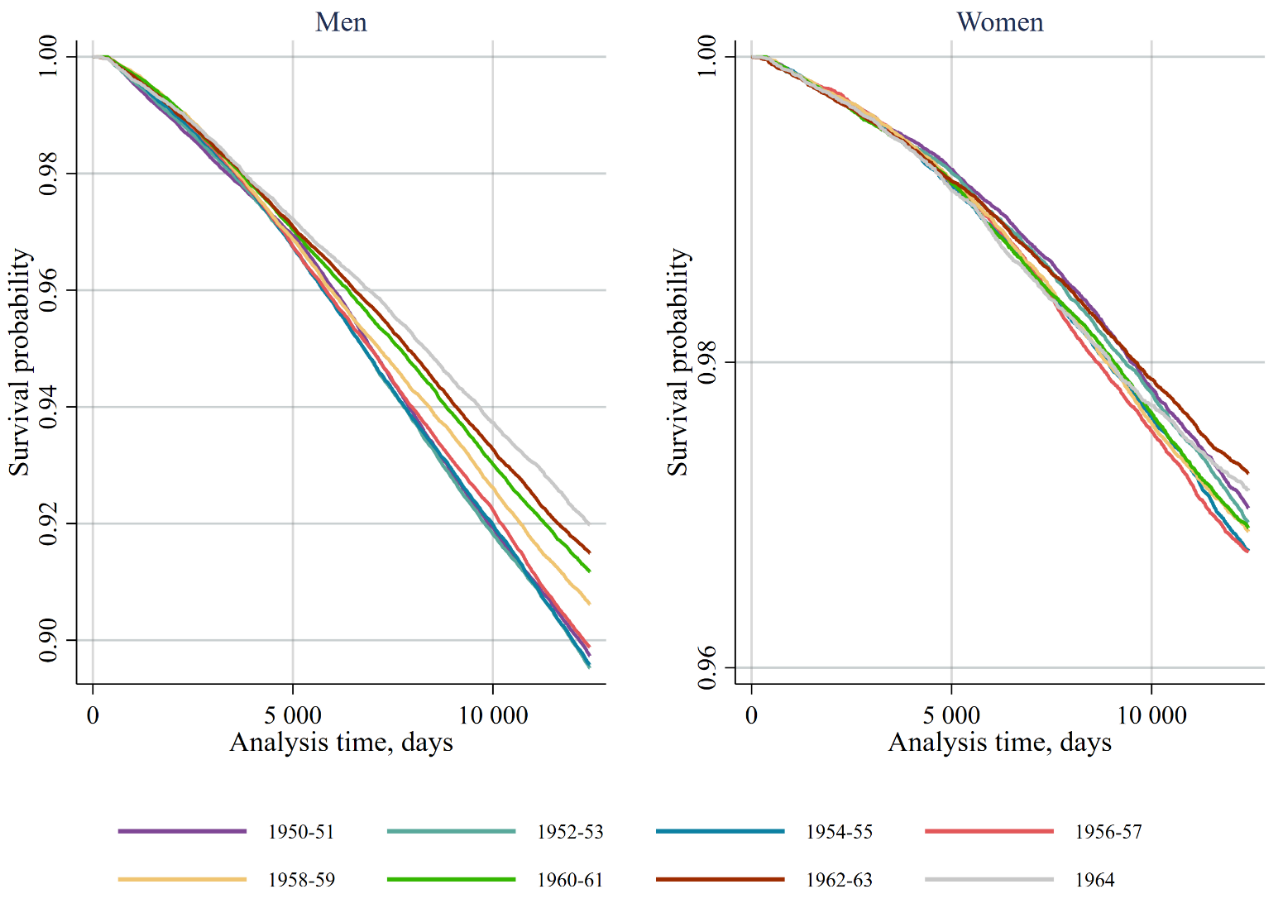

eAppendix 2 *Kaplan-Meier survival curves for alcohol-attributable mortality by gender and birth cohort (1950-1964) during ages 21 to 54 years. Different y-axis scales for men and women.*

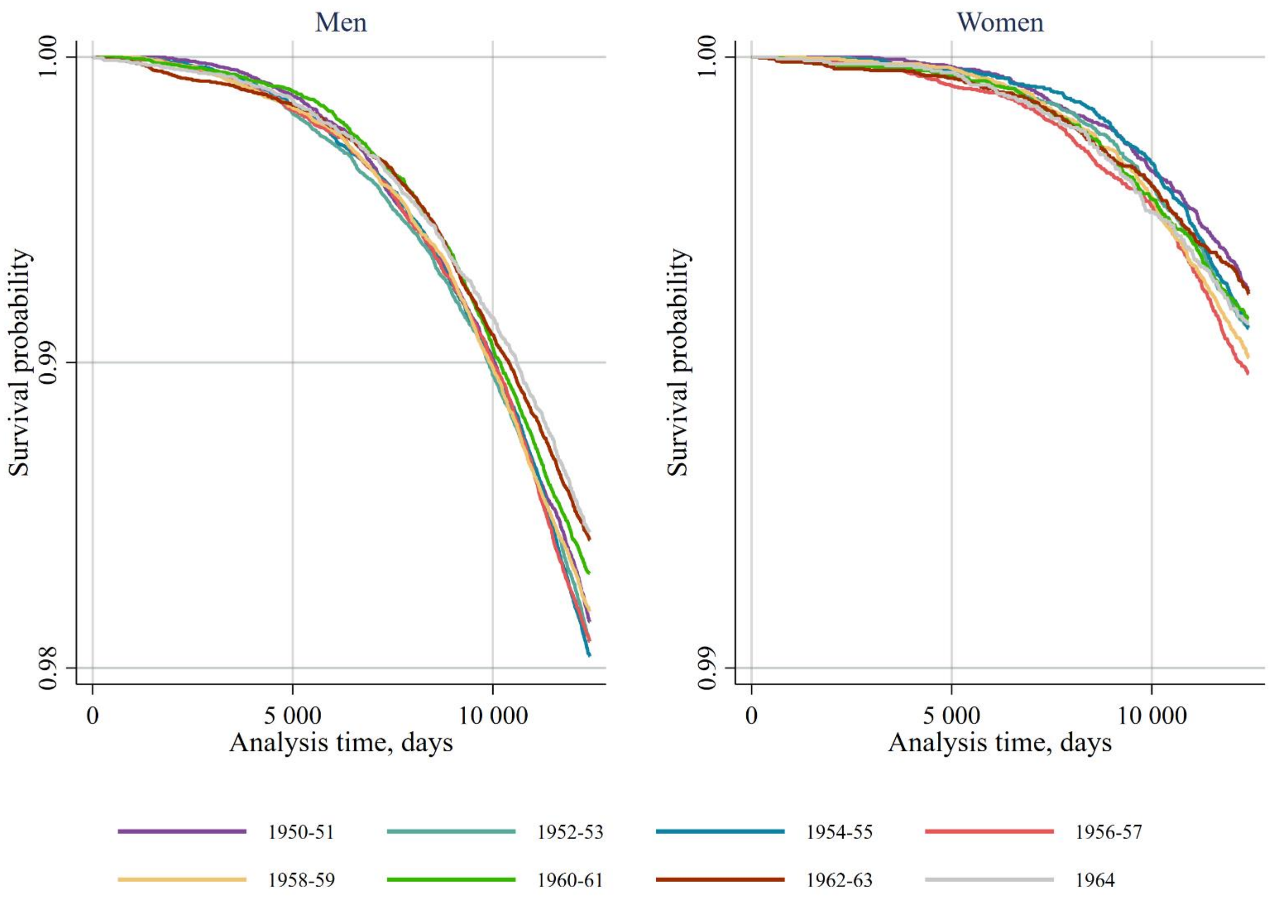

eAppendix 3 *Hazard ratios with 95% confidence intervals for first incidence of alcohol-attributable A: hospitalization and B: mortality between ages 27–54 years by birth cohort (1944-1964). Cohort comparison by exposure to 1969 liberal alcohol reform and exposure to later stricter alcohol policies starting from 1976. Men and women modelled separately. N=855,567 for men, 809,157 for women. Reference category 1957 cohort.*

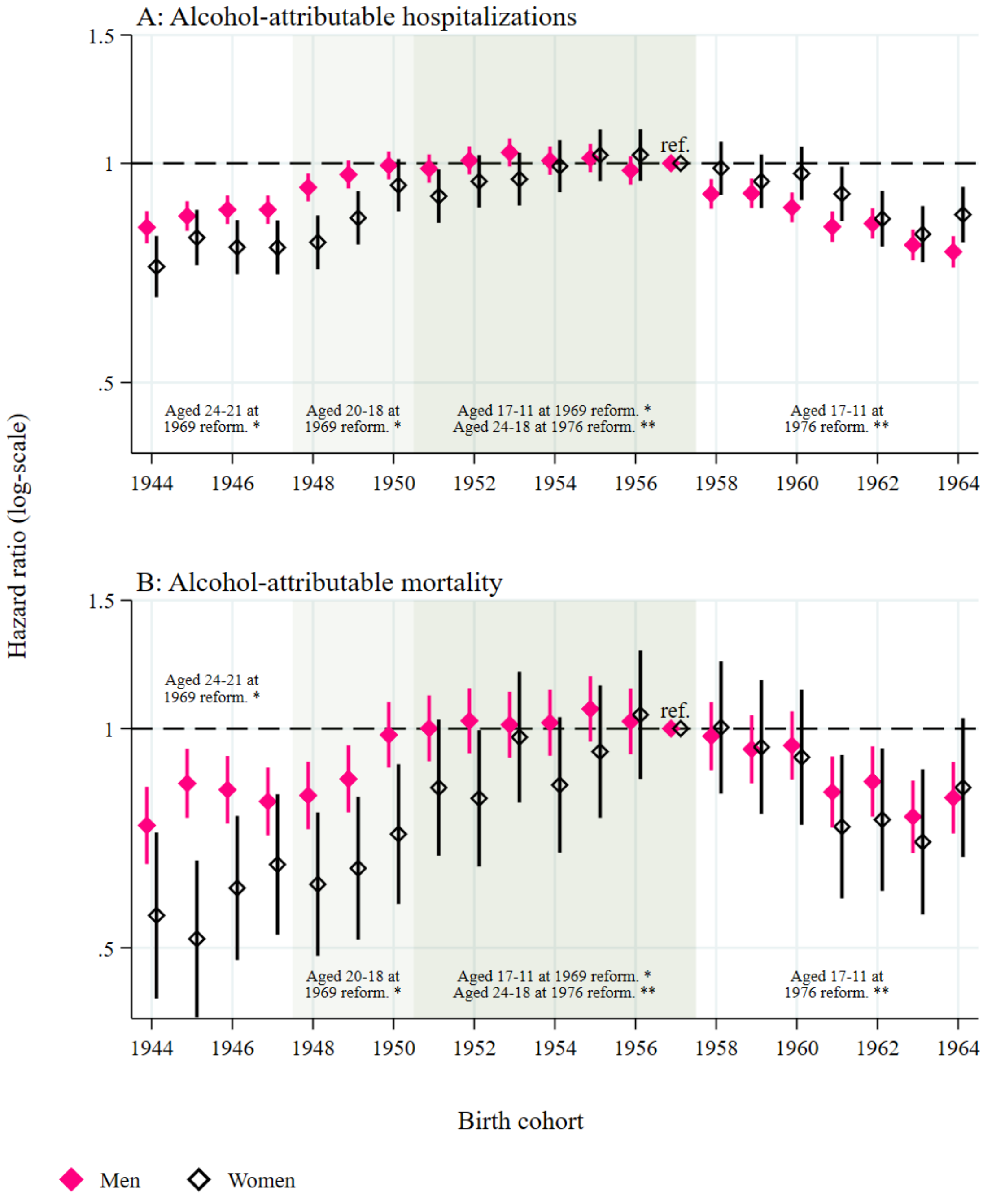

* 1969 liberal reform: MLDA lowered from 21 to 18 years, general improvement on alcohol availability.
** 1976-1977 stringent reform: Alcohol tax hikes and alcohol advertising ban.
Abbreviations: Ref. = reference category; MLDA = minimum legal drinking age.

eAppendix 4 *Hazard ratios with 95% confidence intervals for first incidence of alcohol-attributable A: hospitalization and B: mortality between ages 21–54 years by birth cohort (and age in 1975 when the stricter alcohol policies were implemented). Reference category 1957 cohort (18 years). Excluding the persons who were affected by the education reform laid out during 1972-1977. N=549,150 for men, 520,015 for women.*

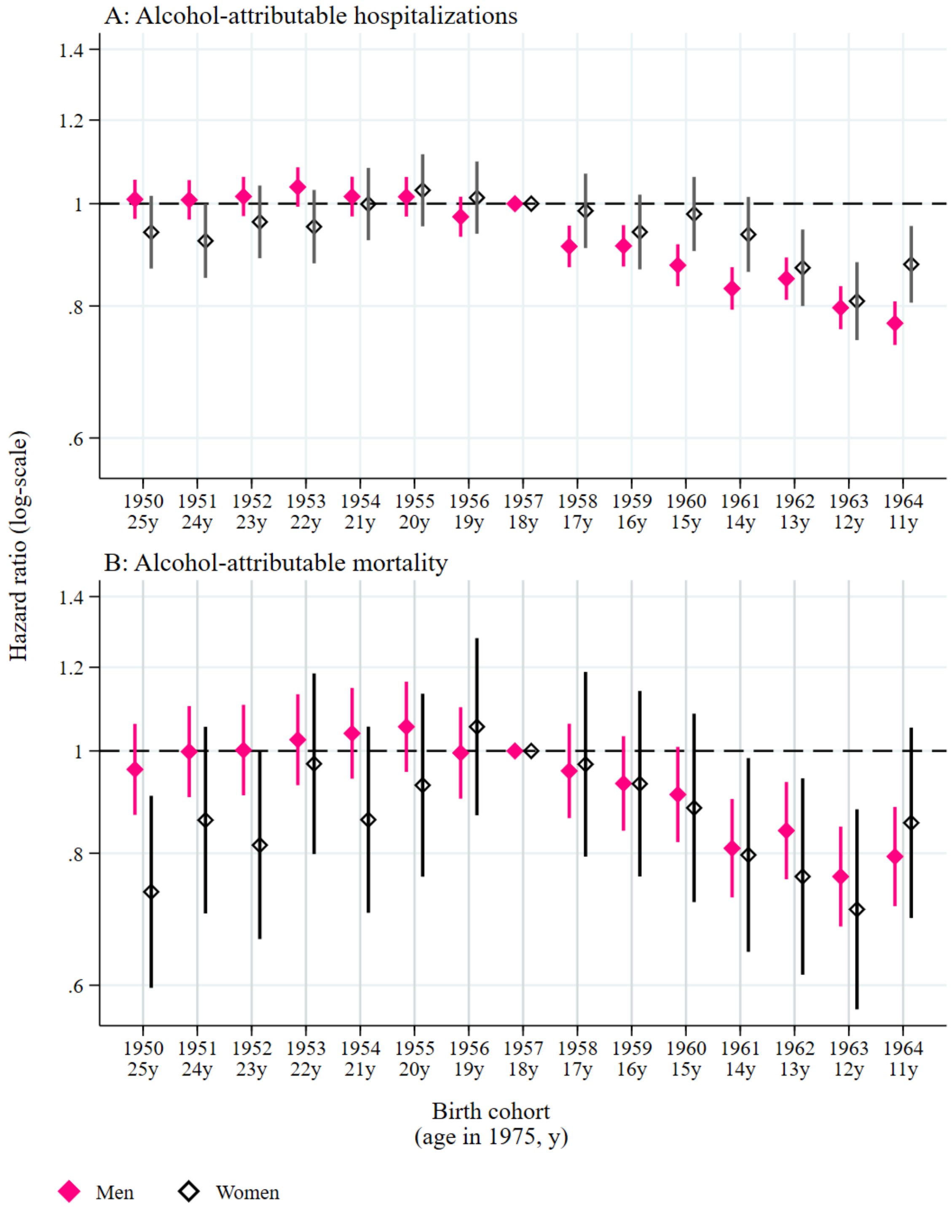

,
